# Effects of Feeding Sunflower Meal and Superdosing Phytase on the Production Traits and Nutrient Digestion of Broiler Chickens

**DOI:** 10.3390/ani16203266

**Published:** 2026-10-16

**Authors:** Kesete Goitom Tewelde, Nikoletta Such, László Pál, Eszter Farkas Péterné, Brigitta Kiss, László Wágner, Károly Dublecz

**Affiliations:** 1 Institute of Physiology and Nutrition, Department of Animal Nutrition and Nutritional Physiology, Hungarian University of Agriculture and Life Sciences, Georgikon Campus, Deák Ferenc Street 16, 8360 Keszthely, Hungary; kesetehac@gmail.com (K.G.T.); such.nikoletta.amanda@uni-mate.hu (N.S.); pal.laszlo@uni-mate.hu (L.P.); peterne.farkas.eszter@uni-mate.hu (E.F.P.); brigitta.kiss@ubm.hu (B.K.); wagner.laszlo@uni-mate.hu (L.W.); 2 Department of Animal Sciences, Hamelmalo Agricultural College, National Higher Education and Research Institute, Keren P.O. Box 397, Eritrea

**Keywords:** broiler chicken, sunflower meal, superdosing phytase, production traits, nutrient digestibility

## Abstract

Sunflower meal (SFM) is an alternative protein source for poultry. It is locally available and cheap, and its protein is rich in methionine and cystine. The high fibre and phytate content of SFM are, however, limitation factors. The aim of this study was to examine the effects of feeding SFM and using superdosing phytase (1500 FTU) on the production performance and nutrient digestibility of broiler chickens. A total of 576-day-old male Ross 308 broilers were fed four iso-nitrogenous and iso-caloric diets: control (C), C with superdosing phytase (CP), SFM, and SFM with superdosing phytase (SFMP). SFM was included at 20% and 30% in the grower and finisher phases, respectively. Neither SFM nor phytase superdosing affected the production traits in the grower phase. However, in the finisher phase, SFM significantly increased body weight gain (BWG) and decreased the feed conversion ratio (FCR). This positive result was also true for the cumulative performance of birds. On day 42, feeding SFM increased fat digestibility and the AMEn content of diets, but decreased the digestion of starch. Superdosing phytase increased both fat and starch digestion, as well as the AMEn and the N-retention of chickens. SFM was found to have no effect on the apparent ileal amino acid (AA) digestibility. Conversely, the phytase treatment significantly increased the absorption of several essential AAs. In conclusion, not only is fibre SFM tolerated by broiler chickens, but it can also improve production traits. Although superdosing phytase positively affects the digestibility of nutrients, it fails to cause changes in the BWG or FCR of chickens.

## Simple Summary

Due to climatic change, the increase in the world population, and the sustainability aspects of animal production, poultry nutrition is facing several challenges. One of the main issues is protein nutrition due the use of more locally available legume seeds and industrial by-products. Extracted sunflower meal (SFM) is an oil industry by-product that is a relatively cheap feedstuff available worldwide, mainly used in animal nutrition. The main constraint of SFM is its high fibre content, and exact information on its maximum inclusion rates in poultry diets is still lacking. Furthermore, exogenous enzymes, like phytase, are used routinely in animal nutrition; they can release more phosphorous, starch, and protein from diets. In this study, the effects of feeding SFM and using superdosing phytase were evaluated with broiler chickens in the grower and finisher phases. Broiler chickens seem to tolerate higher SFM content than that currently used, and phytase improves the digestibility of all nutrients.

## 1. Introduction

Soybean meal is the primary protein source in broiler diets; however, its high cost and import dependency necessitate alternative protein sources. Different agricultural by-products like rapeseed meal, sunflower meal (SFM), DDGS, wheat middling, and wheat bran in poultry diets could serve as alternatives with lower production costs [1].

SFM is a cost-effective by-product of the oil industry, used as a protein source in poultry nutrition. However, its high fibre content can be a limiting factor in young chickens [2,3,4]. In addition, its nutritional value can be significantly affected by the chemical, mechanical, and thermal processing methods used during oil extraction. These treatments may result in complex protein–carbohydrate structures that could further impair nutrient digestion [4]. The NSP content of a high-protein SFM is around 14.6%, which consists of the following: arabinose, 2.8%; xylose, 1.7%; mannose, 1.06%; galactose, 0.97%; glucose, 5.36%; and uronic acids, 2.68% [5]. Despite its anti-nutritive factors, SFM has a considerable potential as an alternative protein source in broiler diets [6,7].

The use of exogenous enzymes improves the value of agro-industrial by-products used as animal feed by enhancing nutrient availability and digestibility and reducing anti-nutritional factors. Animals produce their own digestive enzymes, but these cannot fully break down these materials. Thus, enzyme treatment has become an effective strategy employed to boost productivity and animal performance [8,9]. In the case of SFM, the results from exogenous enzyme supplementation in broiler diets are inconsistent, with some studies showing improved performance [10,11,12], and others reporting no improvement [13].

Defatted sunflower meal typically contains about 3% to 5% phytate [14], and exogenous phytase is used routinely in animal nutrition to improve phosphorous availability. Phytase superdosing (1500–2500 FTU/kg) has also been used in some research, where the focus was to not only release phosphorus but also improve the digestibility of protein, starch, and energy [12,15]. In one study, high-protein SFM was used in broiler diets at 5%, 10%, and 15% inclusion rates, with or without phytase (500 FTU/kg) supplementation. Phytase improved not only the apparent ileal digestibility of nutrients but also the body weight gain (BWG), feed conversion ratio (FCR), and bone traits of broiler chickens [16]. In another broiler trial, however, the addition of microbial phytase (1000 FTU/kg) had no effect on the BWG, FI, or FCR [13]. Therefore, there is a knowledge gap and inconsistency regarding the optimum inclusion rate of SFM and the best exogenous enzyme application [17].

In our previous experiments, different exogenous enzymes and their combinations were evaluated with layer-type pullets and laying hens. The apparent ileal amino acid digestibility was improved only through the use of combinations containing superdosed phytase [18,19]. Considering the high feed consumption rate and quick digesta transit time of broiler chickens, the aim of this study was to examine the effects of superdosing phytase on the production traits and nutrient digestibility of broiler chickens when fed SFM-containing diets.

## 2. Materials and Methods

### 2.1. Birds and Housing

The experiment was conducted at the experimental farm of the Department of Animal Nutrition and Nutritional Physiology, Georgikon Campus, Hungarian University of Agriculture and Life Sciences, following approval from the institutional ethics committee (licence number: MAB-3/2024). A total of 576-day-old male Ross 308 chickens were purchased from a commercial hatchery (Gallus Ltd., Devecser, Hungary) and randomly allocated to 24 pens. Four dietary treatments were applied, with 144 chickens per treatment and six replicates of 24 birds each. Birds were housed on wood shaving litter in an environmentally controlled housing system with a stocking rate of 14 birds/m^2^. Temperature, lighting, and ventilation were identical across pens. Feed and water were provided ad libitum. The lighting program followed the recommendation of Aviagen for Ross 308 [20]. Specifically, a 23L:1D photoperiod with an intensity of 30 lux was applied from day 0 to 7. From day 8 onward, the lighting regimen was set to 20L:4D, and the light intensity was reduced to 10 lux for the remainder of the trial. All birds were vaccinated in the hatchery against infectious bronchitis, Newcastle disease, and infectious bursal disease using vaccines produced by Ceva (Ceva Sante Animale, Libourne, France).

The composition of the experimental diets is shown in Table 1. Four iso-nitrogenous and iso-caloric diets were formulated according to breeder’s recommendations [20]. The dietary treatments consisted of a soybean–wheat–corn-based control diet (C) and sunflower meal-based diet (SFM). These diets contained NSP-degrading enzyme (Natugrain^®^ TS) and phytase (Quantum^®^ Blue 5G; 500 FTU). Since all commercial broiler diets contained phytase, the effects of superdosing phytase was compared to this basal phytase level. The diets with superdosed phytase contained the NSP-degrading enzyme and 1500 FTU phytase (CP and SFMP). The SFM used in this experiment was composed of 35.5% crude protein, 19% crude fibre, and 3.5% crude fat.

During the starter phase (days 0 to 11), all birds were fed a commercial, corn-, wheat-, and soybean meal-based starter diet without SFM. In the grower (day 11 to 24) and finisher phases (day 25 to 36), SFM was incorporated into the diets at 20% and 30%, respectively. SFM mainly replaced soybean meal and corn. To compensate the low-energy content of SFM, a higher amount of sunflower oil was added to the SFM diets. The analysed nutrient content of the experimental diets is shown in Table 2. The measured nutrient contents were close to the predicted values.

### 2.2. Chemical Analysis

The proximate nutrients of the experimental diets were analysed with the standard methods. The neutral detergent fibre (NDF) was determined sequentially as described by Van Soest [21] and expressed on an ash-free basis. The gross energy (GE) of the diets and excreta samples was analysed using a bomb calorimeter (IKA C6000, IKA-Werke GmbH & Co., Breisgau, Germany). The starch content was measured using the polarimetric method according to European Directive 152/2009 [22], and the amino acid content of the feed and digesta samples was analysed using an automatic amino acid analyser (Ingos Amino Acid Analyzer AAA 400, INGOS s.r.o., Prague, Czech Republic) according to ISO standard 13903:2005 [23]. The titanium dioxide (TiO_2_) was determined with a UV spectrophotometer (Jenway 6100, Bibby Scientific Limited, Staffordshire, UK), with absorbance measurements taken at a wavelength of 410 nm, according to [24].

### 2.3. Measurements and Samples

#### 2.3.1. Production Traits

Body weight (BW) and feed intake (FI) were measured at the end of the grower (day 24) and finisher (day 36) phases. Body weight gain (BWG) was calculated by deducting the average initial body weight from the average final body weight. The feed conversion ratio (FCR) was calculated based on grams of feed consumed to produce a gram of weight gain.

#### 2.3.2. Digestibility Trial

On day 36, after excluding outliers deviating significantly from the pen mean (defined as >1.5 SD from the mean), a total of 32 birds (8 animals per treatment) were randomly selected, moved, and placed into 32 individual balance cages. To obtain 8 birds per treatment from the 6 replicate pens, 2 birds were taken from each of the first and second replicate pens and 1 chicken from replicate pens 3–6. Automatic drinkers and feeders were fixed to each cage, and the chickens were fed their finisher diets containing titanium dioxide (TiO_2_) as an indigestible marker at a rate of 5 g/kg. On days 41 and 42, representative excreta samples were collected. On the second day, the daily samples were thoroughly homogenised, frozen, and stored until analysis. On day 42, all animals were euthanised according to national animal welfare legislation (Hungarian Government Decree 40/2013). The abdominal cavity of the birds was opened immediately, and the left caecal sack content and 2/3 of the distal ileum digesta were collected. The gut contents were gently squeezed and poured into Eppendorf tubes (0.5 mL) and stored in a refrigerator at −20 °C before AA analyses.

#### 2.3.3. Digestibility and Metabolizable Energy Calculations

The following equations were used to calculate the digestibility:

$$\text{Nutrient digestibility}\;(\%)=\left(\frac{{\mathrm{Nutr}.}_{\mathrm{diet}}-({\mathrm{Nutr}.}_{\mathrm{excreta}}({\mathrm{TiO}}_{2\;\mathrm{diet}}/{\mathrm{TiO}}_{2\;\mathrm{excreta}}))}{{\mathrm{Nutr}.}_{\mathrm{diet}}}\right)\times 100$$

where

Nutr._diet_ = nutrient content of the diet (%);

Nutr._excreta_ = nutrient content of the excreta (%);

TiO_2 diet_ = TiO_2_ content of the diet (%);

TiO_2 excreta_ = TiO_2_ content of the excreta (%).

$$\text{Nitrogen retention}\;(\%)=\left[\frac{N_{\mathrm{diet}}-{(N_{\mathrm{excreta}}\times (\mathrm{TiO}}_{2\mathrm{diet}}/{\mathrm{TiO}}_{2\;\mathrm{excreta}}))}{N_{\mathrm{diet}}}\right]\times 100$$

where

N_diet_ = nitrogen content of the diet (%);

N_excreta_ = nitrogen content of the excreta (%);

TiO_2 diet_ = TiO_2_ content of the diet (%);

TiO_2 excreta_ = TiO_2_ content of the excreta (%).

$$\mathrm{IAAD}(\%)=\left[\frac{{\mathrm{AA}}_{\mathrm{diet}}-({\mathrm{AA}}_{\mathrm{digesta}}\times ({\mathrm{TiO}}_{2\mathrm{diet}}/{\mathrm{TiO}}_{2\;\mathrm{digesta}}))}{{\mathrm{AA}}_{\mathrm{diet}}}\right]\times 100$$

where

IAAD = Ileal amino acid digestibility (%);

AA_diet_ = Amino acid content of the diet (%);

AA_digesta_ = amino acid content of the ileal digesta (%);

TiO_2 diet_ = TiO_2_ content of the diet (%);

TiO_2 digesta_ = TiO_2_ content of the ileal digesta (%).

$$\mathrm{AME}\left(\mathrm{KJ}/g\right)={\mathrm{GE}}_{\mathrm{diet}}-({\mathrm{GE}}_{\mathrm{excreta}}\times \left(\frac{{\mathrm{TiO}}_{2\;\mathrm{diet}}}{{\mathrm{TiO}}_{2\;\mathrm{excreta}}}\right))$$

where

AME = apparent metabolizable energy (KJ/g);

GE_diet_ = gross energy content of the diet (KJ/g);

GE_excreta_ = gross energy content of the excreta (KJ/g);

TiO_2 diet_ = TiO_2_ content of the diet (g/kg);

TiO_2 excreta_ = TiO_2_ content of the excreta (g/kg).

$$N_{\mathrm{retention}}\left(g/g\right)=N_{\mathrm{diet}}-\left(\frac{{\mathrm{TiO}}_{2\;\mathrm{diet}}}{{\mathrm{TiO}}_{2\;\mathrm{excreta}}}\right)\times N_{\mathrm{excreta}}$$

where

N_retention_ = nitrogen retention (g N/g diet);

N_diet_ = nitrogen content of the diet (g N/g diet);

N_excreta_ = nitrogen content of the excreta (g N/g excreta);

TiO_2 diet_ = TiO_2_ content of the diet (g/kg);

TiO_2 excreta_ = TiO_2_ content of the excreta (g/kg).

AMEn (KJ/g) = AME − (N_retention_ × 34.4)

where

AME_n_ = apparent metabolizable energy corrected to zero N retention;

AME = apparent metabolizable energy (KJ/g);

N_retention_ = nitrogen retention (g N/g diet);

34.4 = constant, the GE value of uric acid (KJ/g N).

### 2.4. Statistical Analysis

All statistical analyses were carried out using software package SPSS 29.0 for Windows, (IBM Corp. in Armonk, NY, USA), with a complete randomised design. The pens were the experimental units for the production traits, using one chicken per cage for the digestibility measurements. The averages of the tested parameters were analysed using two-way ANOVA (2 × 2 factorial, full factorial model) with a general linear model for univariate analysis, using the SFM and enzyme treatments as main factors. Normality and homogeneity of variances were tested using the Shapiro–Wilk and Levene tests, respectively. Statistical significance was declared at *p* < 0.05.

## 3. Results

### 3.1. Production Traits

The production traits of the chickens are shown in Table 3. The dietary inclusion of the SFM and enzyme treatment did not modify the measured parameters during the grower phase. However, during the finisher phase and the entire experimental period (days 0 to 36), feeding SFM significantly improved BWG and FCR. The use of additional phytase did not reveal any effects.

### 3.2. Nutrient Digestibility, Nitrogen Retention, and Metabolizable Energy

The SFM-containing diet and phytase supplementation significantly affected the faecal nutrient digestibility coefficients, the metabolizable energy content of the diets, and the N-retention of the birds (Table 4). The SFM treatment significantly improved the absorption of crude fat and increased dietary AMEn; however, it also decreased starch digestibility. Phytase treatment improved all of the measured parameters. An interaction effect was also observed for fat and starch digestibility; the reason for this is that the effect of phytase on fat digestion was higher in the control group (+2.1%) than with the SFM treatment (+0.8). The opposite phytase effect was found in the case of starch digestibility with SFM (SFM: +3.3% vs. C: +1.8%).

The apparent ileal amino acid (AA) digestibility of the experimental diets is presented in Table 5. The digestibility of AAs ranged between 60% (threonine) and 85% (methionine and arginine). The SFM-containing diet did not modify AA digestibility; however, phytase supplementation significantly increased the digestibility of arginine, lysine, methionine, threonine, valine, and tyrosine. Leucine was the only AA affected negatively by phytase.

## 4. Discussion

### 4.1. Production Traits

The application of alternative protein sources, particularly solvent-extracted sunflower meal (SFM), in poultry nutrition has garnered substantial attention within developing economies due to its local availability and cost-effectiveness. When diets are adequately balanced for energy and essential amino acids—specifically methionine and lysine—SFM can be successfully utilised [25]. Regarding inclusion levels, certain studies have proposed that broiler chickens can tolerate up to 15% SFM without negative effects on growth performance or related parameters [26]. This threshold might be extended further through enzyme supplementation [27,28]. Nevertheless, the literature remains inconsistent; several authors observed diminished growth performance when SFM inclusion reached or exceeded 10–15%, often attributed to the high amounts of crude fibre content and anti-nutritional factors inherent to the hulls [29]. In our previous study with laying hens, however, we established that SFM could be incorporated at substantially higher levels without compromising nutrient utilisation, demonstrating that dietary inclusion of up to 30% SFM did not adversely affect the ileal digestibility of essential amino acids [30]. Building upon these earlier findings and extending the concept to meat-type poultry, the present treatments containing 20% SFM during the grower phase and 30% SFM during the finisher phase improved the production performance. This enhancement may be explained by the advanced physiological development of the birds’ gastrointestinal tract in the later growth phases, where the additional insoluble fibre can stimulate gizzard function and development, thereby optimising feed intake and digesta retention times.

To improve nutrient utilisation and increase available energy, enzyme supplements are commonly applied. As monogastric farm animals, poultry and swine struggle to hydrolyse phytate-bound P into inorganic P due to the lack of endogenous phytase secretion [31,32]. Phytic acid has a strong binding affinity to soluble proteins, amino acids, minerals, and starch at low pH, which reduces the digestibility of these complex nutrients [33]. Hence, besides the improvement on phosphorus digestibility, the addition of phytase favours the availability of nutrients that would otherwise be complexed to the phytic acid molecule [12]. In addition to NS-degrading enzyme supplementation, phytase superdosing can provide additional phosphoric benefits [34]. Several studies have examined the beneficial effects of high doses of phytase on production parameters, but only a few research results are available on sunflower meal-containing diets. Lee et al. [35] used a corn–soybean-based diet in a 42-day feeding trial and demonstrated the beneficial effect of phytase superdosing (1500 FTU/kg). Although phytase superdosing increased weight gain and reduced the feed conversion ratio (FCR) at all P levels (*p* < 0.05), the response was more pronounced when low-P diets were fed. Similarly, Paul et al. [36] found that phytase superdosing (2000 FTU/kg feed) of a maize-soybean meal-based diets resulted in better feed efficiency in broiler chickens compared to the control or to an antimicrobial growth promoter treatment. Only one study has examined the phytase effects on sorghum-based diets. In that trial, the interactions among xylanase, protease, and phytase superdosing interactions on broiler performance were evaluated. According to their results, phytase superdosing increased the BW and FI at day 42 (*p* < 0.05) and xylanase improved FCR (*p* < 0.05), although no additive effects were observed among the three enzymes [37]. Recently, Léo et al. [15] evaluated the effects of phytase superdosing on the performance, carcass yield, pectoral myopathies, and bone mineral composition of broiler chickens, as well as their dietary metabolizable energy content. Their experiment involved corn, soybean meal-, meat-, and bone meal-based diets and phytase supplementation (2000 FTU/kg), which resulted in higher weight gain and better FCR. Consequently, high-dose phytase can be an effective strategy even in the case of diets that contain mixed phosphorus sources, including animal-derived ingredients.

In our previous experiment, high- and low-fibre SFM was fed at 20% and 30% inclusion rates to broiler chickens. The diets contained a commercial dose of phytase and NSP-degrading enzymes. No significant SFM treatment effects were found in the grower phase (day 11–day 24), but high-fibre SFM at 30% inclusion significantly increased the BWG in the finisher phase (day 25–day 38). However, in this experiment, we did not use phytase superdosing [7]. These results are consistent with those of the current experiment, where 20% SFM failed to affect the production traits, but SFM at the 30% inclusion level improved both the BWG and FCR. The reason for this could be that older broilers have a higher feed intake capacity, and dietary SFM may stimulate gizzard development, which regulates feed intake and nutrient digestibility in a positive manner [38]. Furthermore, older birds were more capable of tolerating dietary fibre, enabling them to maintain efficient nutrient utilisation [6]. In conclusion, the findings of this study indicate that SFM can be included in the broiler grower diets up to 20% without compromising production traits, while feeding 30% SFM in the finisher phase can even improve the growth rate and FCR of the chickens.

### 4.2. Nutrient Digestibility, Nitrogen Retention, and Metabolizable Energy

In this experiment, 30% SFM significantly increased dietary AMEn and fat digestibility, yet decreased starch digestion. The reason for these contrasting results could be that the diluting effect of fibre hinders the efficiency of α-amylase [7]. Moreover, micelle formation in fat digestion is less sensitive to fibre [39]. Furthermore, the fibre content of SFM predominantly comprises insoluble fractions (cellulose and lignin), which do not increase digesta viscosity, unlike soluble non-starch polysaccharides [40], thereby maintaining an optimal environment for micelle formation and lipase activity [9].

In contrast to the production parameters, superdosing phytase significantly increased the faecal nutrient digestibility of all nutrients and the N-retention of the chickens. The benefits of exogenous enzymes are usually higher in young flocks [9], although the response is occasionally more pronounced in the finisher phase. Phytates affect not only the availability of phosphorous but also the protein digestion and energy utilisation in poultry [41,42]. Fernandes et al. [12] observed the benefits of increasing exogenous phytase activity from 1000 to 1500 FTU on the digestibility coefficients and metabolizable energy content of corn–soybean meal-based diets. The reason for improvement could be that higher doses of phytase may result in a more efficient release of the phytate–starch and phytate–protein linkages [34]. In our trial, although superdosing phytase significantly increased nutrient digestibility, it did not result in improvements in the production traits. The reason for this apparent contradiction could be that the basic absorption rate of fat, starch, and protein supplied enough nutrients and covered the chicken’s requirements.

In poultry nutrition, ileal digestibility is the preferred measure for estimating the AA digestibility of feed ingredients and the AA requirements of birds [43].

In this, study, 30% SFM had no effect on the digestion of amino acids. Similarly to our findings, the highest amino acid digestibility coefficients of SFM-containing diets were found for arginine and the lowest for threonine in other experiments [5]. The ileal amino acid digestibility of sunflower meal was investigated by Ullah et al. though an experiment with broiler chickens [44]. They found that the average amino acid digestibility of sunflower meal was lower than that of soybean meal (85.1 vs. 77.1%), due to the higher fibre content of SFM, which showed a significant negative correlation with amino acid digestibility. Enzyme supplementation was, however, not used in that experiment.

Only a few studies have examined the effect of phytase superdosing on the ileal amino acid digestibility of sunflower meal in broiler chickens. In the study of Wolfrun et al. [45], the phytase effect on amino acid digestibility was investigated with broiler chickens fed soybean meal, rapeseed meal, sunflower meal, and rice bran-containing diets. According to their findings, the phytase supplementation at 1500 FTU/kg did not affect the prececal AA digestibility even with increasing oilseed meal–rice bran incorporation levels. Another study examined the phytase effects on ileal AA digestibility and phytate breakdown when a soybean meal, a sunflower meal (300 g/kg), or rapeseed meal-based diets were fed. The prececal phytate disappearance was significantly affected by interactions between the oilseed meal inclusion levels and phytase supplementation. Overall, prececal phytate disappearance was higher in soybean meal (SBM) diets (52%) than in SFM diets (38%) and intermediate in rapeseed meal (RSM) diets (43%). The average increase in AA digestibility was 7% in rapeseed meal, whereas it was 3% in SFM and 1% in SBM. Because AA digestibility without phytase was lower in RSM and SFM than in SBM, the phytase supplementation reduced the differences in AA digestibility levels among oilseed meals [46]. In our trial the average improvement in essential AA digestibility was 2.6% in the control and 2.8% in the SFM group. The results are however not entirely comparable because of the differences in diet compositions, the nutrient content of SFM, and the animal trial methodology (apparent versus standardised digestibility). The selective phytase effect on AA digestibility suggests that phytase superdosing releases peptides that are relatively rich in arginine, lysine, methionine, threonine, valine, and tyrosine. The exact mechanism behind these results, including the negative phytase effect on leucine absorption, requires further investigation.

## 5. Conclusions

From the results of this experiment, it can be concluded that broiler chickens can tolerate 20% SFM in the grower period, but only 30% SFM in the finisher diets could even improve the weight gain of the birds. The reason behind this result could be the proper gizzard function and all of its positive aspects on feed intake regulation and enzyme secretion. SFM improves fat digestion, increases dietary AMEn, depresses starch digestibility, but does not modify AA digestibility or N-retention. Superdosed phytase improves the digestibility of fats, starch, and the ileal absorption rate of arginine, lysine, methionine, threonine, valine and tyrosine, but these improvements could not have been translated to the production traits.

## Figures and Tables

**Table 1:** Composition of the experimental diets (g/kg).

Ingredients	Starter	Grower				Finisher			
		C	CP	SFM	SFMP	C	CP	SFM	SFMP
Corn	392.0	472.0	472.0	363.0	363.0	533.0	533.0	370.0	370.0
Wheat	100.0	100.0	100.0	100.0	100.0	100.0	100.0	100.0	100.0
Extracted soybean meal	400.0	324.0	324.0	193.0	193.0	267.0	267.0	71.0	71.0
Extracted sunflower meal	0.0	0.0	0.0	200.0	200.0	0.0	0.0	300.0	300.0
Sunflower oil	55.0	56.0	56.0	94.0	94.0	54.0	54.0	111.0	111.0
Monocalcium phosphate	16.0	15.0	15.0	15.0	15.0	13.0	13.0	14.0	14.0
Limestone	18.0	15.0	15.0	14.0	14.0	13.0	13.0	11.0	11.0
Premix ^1^	5.0	5.0	5.0	5.0	5.0	5.0	5.0	5.0	5.0
Salt	3.0	3.0	3.0	3.0	3.0	3.0	3.0	3.0	3.0
Sodium bicarbonate	1.0	1.0	1.0	1.0	1.0	1.0	1.0	1.0	1.0
Lysine	4.0	4.0	4.0	6.0	6.0	5.0	5.0	8.0	8.0
Methionine	4.0	3.0	3.0	3.0	3.0	3.0	3.0	3.0	3.0
Threonine	1.0	1.0	1.0	2.0	2.0	2.0	2.0	2.0	2.0
Valine	1.0	0.5	0.5	0.5	0.5	1.0	1.0	1.0	1.0
Natugrain^®^ TS ^2^	0.1	0.1	0.1	0.1	0.1	0.1	0.1	0.1	0.1
Phytase ^3^	0.1	0.1	0.3	0.1	0.3	0.1	0.3	0.1	0.3
Coccidiostats ^4^	0.6	0.6	0.6	0.6	0.6	0.0	0.0	0.0	0.0
Total (g)	1000	1000	1000	1000	1000	1000	1000	1000	1000

C—control; CP—C + phytase; SFM—sunflower meal; SFMP—SFM + phytase. ^1^ Supplied by Agrofeed Ltd. (Győr; Hungary). The active ingredients contained in the premix were as follows (per kg of complete diet): vitamin A—10,000 IU; vitamin D3—3000 IU; vitamin E—30 mg; menadione—2 mg; thiamine—2.18 mg; riboflavin—6 mg; pyridoxin HCl—3 mg; cyanocobalamin—0.02 mg; niacin—31.27 mg; pantothenic acid—9.13 mg; folic acid—1.5 mg; biotin—0.15 mg; betaine—150 mg; BHT—0.40 mg; BHA—0.40 mg; citric acid—0.36 mg; Zn (as ZnO)—40 mg; Zn (as 3b607)—40 mg; Cu (as 3b413)—10 mg; Fe (as FeSO4H2O)—50 mg; Mn (as MnO)—50 mg; Mn (as 3b506)—50 mg; I [as Ca(IO3)2]—1.5 mg; Se (as C5H11NO2Se)—0.2 mg. ^2^ Natugrain^®^ TS, BASF endo-1,4-beta-xylanase (5.60 TXU/kg) and endo-1,4-beta-glucanase (2.50 TGU/kg). ^3^ Phytase (Quantum^®^ Blue 5G, 6-phytase, 5000 FTU/g) as 500 FTU/kg diet in the C + SFM dietary group and 1500 FTU/kg diet in the CP + SFMP dietary group. ^4^ Coccidiostat—starter phase: Maxiban TM G160—600 g/t; grower phase: Elancoban—600 g/t.

**Table 2:** Measured and calculated nutrient content of the experimental diets (%).

Nutrients		Grower				Finisher			
	Starter	C	CP	SFM	SFMP	C	CP	SFM	SFMP
AMEn (MJ/kg) ^1^	12.60	13.00	13.00	13.00	13.00	13.20	13.20	13.20	13.20
Dry matter	89.81	89.51	89.93	90.68	90.48	89.30	88.71	89.88	89.71
Crude protein	24.47	19.91	20.06	20.70	20.62	18.78	18.20	18.71	18.86
Crude fat	7.31	7.28	7.30	10.25	10.75	7.52	7.41	12.00	12.44
Ash	7.27	5.99	5.91	6.43	6.42	6.43	5.79	6.39	6.71
Calcium	1.04	0.97	0.99	1.01	0.98	0.91	0.88	0.89	0.92
Phosphorus	0.77	0.70	0.72	0.74	0.74	0.64	0.64	0.65	0.66
Starch	32.77	40.17	40.32	32.72	32.42	41.64	42.14	31.84	30.73
Crude fibre	4.17	4.40	3.98	9.22	9.30	4.75	4.63	9.78	9.86
NDF ^2^	13.78	13.93	13.85	15.72	15.79	14.02	14.29	16.72	16.61
IDF ^3^	13.17	12.59	12.37	15.25	15.41	11.47	11.52	16.67	16.32
SDFP ^4^	3.69	3.21	3.44	3.65	3.53	2.93	2.90	4.07	3.89
Na^+^ (mEq/kg)	83.34	84.41	84.41	87.89	87.89	84.47	84.47	89.73	89.73
K^+^ (mEq/kg)	314.48	276.25	276.25	213.34	213.34	243.95	243.95	150.07	150.07
Cl^−^ (mEq/kg)	63.63	63.53	63.53	62.14	62.14	63.40	63.40	61.35	61.35
DEB ^5^ (mEq/kg)	335.19	297.13	297.13	239.09	239.09	265.02	265.02	178.46	178.46
ARG ^6^	1.60	1.29	1.30	1.41	1.44	1.17	1.15	1.36	1.34
HIS	0.63	0.50	0.50	0.55	0.53	0.48	0.47	0.45	0.47
ILE	1.01	0.83	0.82	0.91	0.84	0.75	0.73	0.71	0.71
LEU	1.91	1.61	1.61	1.49	1.55	1.52	1.49	1.35	1.40
LYS	1.55	1.25	1.24	1.27	1.27	1.18	1.17	1.18	1.17
MET	0.71	0.55	0.56	0.58	0.58	0.52	0.52	0.58	0.58
PHE	1.20	0.95	0.98	0.97	0.98	0.90	0.87	0.85	0.87
THR	0.99	0.80	0.85	0.93	0.96	0.86	0.83	0.88	0.87
VAL	1.22	0.96	0.94	0.98	1.01	0.94	0.91	0.96	0.97
CYS	0.37	0.31	0.31	0.33	0.33	0.30	0.29	0.32	0.31
TYR	0.80	0.65	0.66	0.62	0.62	0.59	0.59	0.49	0.53

C—control; CP—C + Phytase; SFM—sunflower meal; SFMP—SFM + Phytase. ^1^ Nitrogen-corrected apparent metabolizable energy (calculated value); ^2^ neutral detergent fibre; ^3^ insoluble dietary fibre; ^4^ soluble dietary fibre precipitable; ^5^ dietary electrolyte balance; ^6^ ARG—arginine; HIS—histidine; ILE—isoleucine; LEU—leucine; LYS—lysine; MET—methionine; PHE—phenylalanine; THR—threonine; VAL—valine; CYS—cystine; TYR—tyrosine.

**Table 3:** Effects of sunflower meal and extra phytase on the production traits of broiler chickens.

	Feed (F)	Phytase (P)	FI (g)	BWG (g)	FCR (g/g)
Grower phase (Days 11–24)	C	−	1197.8 ± 15.97	856.3 ± 20.73	1.40 ± 0.00
		+	1185.8 ± 29.45	853.9 ± 22.45	1.38 ± 0.02
	SFM	−	1188.7 ± 9.87	897.6 ± 9.57	1.33 ± 0.02
		+	1190.0 ± 7.15	880.2 ± 12.84	1.37 ± 0.03
Finisher phase (Days 25–36)	C	−	1639.6 ± 19.43	974.8 ± 19.74	1.67 ± 0.05
		+	1743.0 ± 80.48	1059.5 ± 14.27	1.63 ± 0.08
	SFM	−	1710.3 ± 26.11	1168.6 ± 24.40	1.47 ± 0.02
		+	1698.9 ± 6.53	1148.6 ± 37.46	1.50 ± 0.03
Overall mean (Days 11–36)	C	−	2837.4 ± 20.50	1831.1 ± 38.26	1.57 ± 0.03
		+	2928.8 ± 99.85	1913.4 ± 31.63	1.53 ± 0.04
	SFM	−	2901.6 ± 32.63	2066.3 ± 30.01	1.42 ± 0.03
		+	2874.9 ± 22.20	2028.8 ± 48.41	1.44 ± 0.02
Grower phase	Feed	C	1191.8	855.1	1.39
		SFM	1189.4	889.0	1.35
	Phytase	−	1193.2	877.0	1.37
		+	1187.9	867.0	1.38
		SEM	10.077	8.436	0.009
Finisher phase	Feed	C	1691.3	1017.2 ^b^	1.65 ^a^
		SFM	1704.6	1158.6 ^a^	1.48 ^b^
	Phytase	−	1675.0	1071.7	1.57
		+	1721.0	1104.1	1.57
		SEM	26.655	17.680	0.033
Overall mean (Days 11–36)	Feed	C	2883.1	1872.2 ^b^	1.55 ^a^
		SFM	2888.2	2047.5 ^a^	1.43 ^b^
	Phytase	−	2869.5	1948.7	1.49
		+	2901.9	1971.1	1.49
		SEM	32.286	22.981	0.021
*p*-values	Grower phase	Feed	0.897	0.064	0.099
		Phytase	0.778	0.571	0.732
		F × P	0.724	0.667	0.310
	Finisher phase	Feed	0.788	0.001	0.004
		Phytase	0.356	0.219	1.000
		F × P	0.253	0.053	0.519
	Overall mean	Feed	0.929	0.001	0.002
		Phytase	0.577	0.559	0.885
		F × P	0.314	0.129	0.418

FI—feed intake; BW—body weight; BWG—body weight gain; FCR—feed conversion ratio; F × P—feed and phytase interaction effect. ^a,b^ Means with different superscripts in the same column are significantly different (*p* < 0.05). Values in the upper section represent the treatment means ± standard error of the mean (SEM); SEM in the lower section represents the pooled standard error of the mean for the main effects.

**Table 4:** Effects of sunflower meal and superdosed phytase on the faecal nutrient digestibility, dietary metabolizable energy, and nitrogen retention of broiler chickens.

Feed	Phytase	Fat (%)	Starch (%)	AMEn (kJ/g)	N-Retention (%)
C	−	90.2 ± 0.27	86.9 ± 0.22	13.5 ± 0.05	80.1 ± 0.66
	+	92.3 ± 0.25	88.7 ± 0.25	13.6 ± 0.09	81.7 ± 0.79
SFM	−	94.3 ± 0.07	84.3 ± 0.38	14.1 ± 0.07	79.7 ± 0.54
	+	95.1 ± 0.10	87.6 ± 0.28	14.4 ± 0.08	80.6 ± 0.29
Feed	C	91.2 ^b^	87.8 ^a^	13.5 ^b^	80.9
	SFM	94.7 ^a^	86.0 ^b^	14.2 ^a^	80.2
Phytase	−	92.2 ^b^	85.6 ^b^	13.8 ^b^	79.9 ^b^
	+	93.7 ^a^	88.2 ^a^	14.0 ^a^	81.2 ^a^
	SEM	0.36	0.33	0.08	0.30
*p*-value	Feed	0.001	0.001	0.001	0.177
	Phytase	0.001	0.001	0.019	0.031
	F × P	0.003	0.014	0.262	0.555

C—control; SFM—sunflower meal; F × P—feed and phytase interaction; AMEn—apparent metabolizable energy corrected for zero nitrogen retention. ^a,b^ Means with different superscripts in the same column are significantly different (*p* < 0.05). Values in the upper section represent the treatment means ± standard error of the mean (SEM); SEM in the lower section represents the pooled standard error of the mean for main effects.

**Table 5:** Digestibility of essential amino acids as affected by sunflower meal and superdosing phytase (%).

Feed	Phytase	ARG	HIS	ILE	LEU	LYS	MET	PHE	THR	VAL	CYS	TYR
C	−	79.1 ± 0.74	77.2 ± 1.11	78.0 ± 1.15	84.6 ± 0.79	70.0 ± 1.55	82.7 ± 0.47	82.3 ± 0.85	60.4 ± 1.36	75.6 ± 0.95	75.6 ± 0.93	70.7 ± 1.32
	+	83.7 ± 0.53	78.0 ± 0.94	80.0 ± 0.73	83.4 ± 0.58	74.2 ± 0.86	84.8 ± 0.48	82.9 ± 0.59	68.2 ± 1.19	77.7 ± 0.85	73.5 ± 1.17	78.6 ± 1.22
SFM	−	79.3 ± 0.56	76.1 ± 0.85	80.1 ± 0.77	84.5 ± 0.55	69.4 ± 1.06	84.1 ± 0.67	82.7 ± 0.62	58.9 ± 1.04	75.3 ± 0.85	73.6 ± 0.80	70.0 ± 1.12
	+	85.1 ± 0.32	77.9 ± 0.78	80.2 ± 0.46	81.7 ± 0.51	74.1 ± 0.48	85.0 ± 0.52	82.5 ± 0.76	66.8 ± 0.88	78.4 ± 0.73	74.1 ± 0.61	78.9 ± 0.75
Feed	C	81.4	77.6	79.0	84.0	72.0	83.7	82.6	64.3	76.7	74.5	74.7
	SFM	82.2	77.0	80.1	83.1	71.7	84.6	82.6	62.8	76.8	73.8	74.5
Phytase	−	79.2 ^b^	76.6	79.0	84.6 ^a^	69.7 ^b^	83.4 ^b^	82.5	59.6 ^b^	75.5 ^b^	74.6	70.4 ^b^
	+	84.5 ^a^	77.9	80.0	82.5 ^b^	74.1 ^a^	84.9 ^a^	82.7	67.5 ^a^	78.0 ^a^	73.8	78.8 ^a^
	SEM	0.55	0.47	0.43	0.36	0.66	0.31	0.34	0.91	0.47	0.46	0.94
*p*-value	Feed	0.161	0.542	0.189	0.200	0.751	0.133	0.974	0.224	0.826	0.447	0.862
	Phytase	0.001	0.176	0.220	0.003	0.001	0.008	0.745	0.001	0.006	0.425	0.001
	F × P	0.321	0.615	0.265	0.214	0.870	0.262	0.617	0.959	0.593	0.177	0.703

C—control; SFM—sunflower meal; F × P—feed and phytase interaction; ARG—arginine; HIS—histidine; ILE—isoleucine; LEU—leucine; LYS—lysine; MET—methionine; PHE—Phenylalanine; THR—threonine; VAL—valine; CYS—cystine; TYR—tyrosine. ^a,b^ Means with different superscripts in the same column are significantly different (*p* < 0.05). Values in the upper section represent the treatment means ± standard error of the mean (SEM); SEM in the lower section represents the pooled standard error of the mean for main effects.

## Data Availability

All data generated or analysed during this study are included in this published article.

## References

[1] Njeri F.M., Patterson R., Gachuiri C.K., Kiarie E.G. (2023). Effects of Pretreating Wheat Middlings and Sunflower Meal with Fiber Degrading Enzymes on Components Solubilization and Utilization in Broiler Chickens. Transl. Anim. Sci..

[2] Lannuzel C., Smith A., Mary A.L., Della Pia E.A., Kabel M.A., de Vries S. (2022). Improving Fiber Utilization from Rapeseed and Sunflower Seed Meals to Substitute Soybean Meal in Pig and Chicken Diets: A Review. Anim. Feed Sci. Technol..

[3] Singh A.K., Kim W.K. (2021). Effects of Dietary Fiber on Nutrients Utilization and Gut Health of Poultry: A Review of Challenges and Opportunities. Animals.

[4] Ditta Y.A., King A.J. (2017). Recent Advances in Sunflower Seed Meal as an Alternate Source of Protein in Broilers. Worlds Poult. Sci. J..

[5] Waititu S.M., Kim J.W., Sanjayan N., Leterme P., Nyachoti C.M. (2018). Metabolizable Energy and Standardized Ileal Digestible Amino Acid Contents of a High-Protein Sunflower Meal Fed to Broiler Chicks. Can. J. Anim. Sci..

[6] Munawar Z., Amjid S., Ramzan F., Rafique A., Hassan S., Anwar U., Mehmood M., Farooq U., Khalid M.F., Mustafa R. (2025). Effects of Partial Soybean Meal Replacement with Sunflower Meal and Non-Starch Polysaccharide Degrading Enzymes Supplementation on Broiler Growth Performance, Nutrient Digestibility, and Gut Morphology. Vet. World.

[7] Tewelde K.G., Kiss B., Csiszér T., Pál L., Such N., Bartos Á., Dublecz K. (2026). Feeding Low- and High-Fibre Sunflower Meal to Broiler Chickens—Effects of Inclusion Rate and Age of Birds on the Production Traits, Carcass Composition, Nutrient Digestibility, Gut Viscosity, and Caecal Short-Chain Fatty Acid Content. Animals.

[8] Ojha B.K., Singh P.K., Shrivastava N. (2018). Enzymes in the Animal Feed Industry. Enzymes in Food Biotechnology.

[9] Ravindran V. (2013). Feed Enzymes: The Science, Practice, and Metabolic Realities. J. Appl. Poult. Res..

[10] Waititu S.M., Sanjayan N., Hossain M.M., Leterme P., Nyachoti C.M. (2018). Improvement of the Nutritional Value of High-Protein Sunflower Meal for Broiler Chickens Using Multi-Enzyme Mixtures. Poult. Sci..

[11] Yaqoob M.U., Yousaf M., Imran S., Hassan S., Iqbal W., Zahid M.U., Ahmad N., Wang M. (2022). Effect of Partially Replacing Soybean Meal with Sunflower Meal with Supplementation of Multienzymes on Growth Performance, Carcass Characteristics, Meat Quality, Ileal Digestibility, Digestive Enzyme Activity and Caecal Microbiota in Broilers. Anim. Biosci..

[12] Fernandes J.I.M., Horn D., Ronconi E.J., Buzim R., Lima F.K., Pazdiora D.A. (2019). Effects of Phytase Superdosing on Digestibility and Bone Integrity of Broilers. J. Appl. Poult. Res..

[13] Ciurescu G., Vasilachi A., Grigore D., Grosu H. (2019). Growth Performance, Carcass Traits, and Blood Biochemistry of Broiler Chicks Fed with Low-Fibre Sunflower Meal and Phytase. S. Afr. J. Anim. Sci..

[14] Krum N. (2023). A New Approach for Processing and Use of Sunflower Meal. Bulg. J. Agric. Sci..

[15] de Léo R.A.R., Valentim J.K., Ferreira R.d.S., Almeida A.A., de Farias M.R.S., Hannas M.I. (2026). Phytase Superdosing in Broiler Diets Containing Animal-Derived Protein: Effects on Performance, Bone Mineralisation and Nutrient Metabolisability. Br. Poult. Sci..

[16] Kim J.W., Sanjayan N., Leterme P., Nyachoti C.M. (2019). Relative Bioavailability of Phosphorus in High-Protein Sunflower Meal for Broiler Chickens and Effects of Dietary Phytase Supplementation on Bone Traits, Growth Performance, and Apparent Ileal Digestibility of Nutrients. Poult. Sci..

[17] Mbukwane M.J., Nkukwana T.T., Plumstead P.W., Snyman N. (2022). Sunflower Meal Inclusion Rate and the Effect of Exogenous Enzymes on Growth Performance of Broiler Chickens. Animals.

[18] Such N., Mezőlaki Á., Tewelde K.G., Pál L., Horváth B., Poór J., Dublecz K. (2024). Feeding Sunflower Meal with Pullets and Laying Hens Even at a 30% Inclusion Rate Does Not Impair the Ileal Digestibility of Most Amino Acids. Front. Vet. Sci..

[19] Mezolaki Á., Such N., Wágner L., Rawash M., Tewelde K., Pál L., Poór J., Dublecz K. (2023). Evaluation the Nutrient Composition of Extracted Sunflower Meal Samples, Determined with Wet Chemistry and near Infrared Spectroscopy. J. Cent. Eur. Agric..

[20] Aviagen (2018). Roos Broiler Management Handbook.

[21] Van Soest P.J., Robertson J.B., Lewis B.A. (1991). Methods for Dietary Fiber, Neutral Detergent Fiber, and Nonstarch Polysaccharides in Relation to Animal Nutrition. J. Dairy Sci..

[22] European Commission (2009). Commision Regulation (EC) No 152/2009 of 27 January 2009. Off. J. Eur. Union.

[23] Llames, Fontaine (1994). Commission Directive 98/64/EC of 3 September 1998, Establishing Community Methods of Analysis for the Determination of Amino Acids, Crude Oils and Fats, and Olaquindox in Feeding Stuffs. J. AOAC Int..

[24] Short F.J., Gorton P., Wiseman J., Boorman K.N. (1996). Determination of Titanium Dioxide Added as an Inert Marker in Chicken Digestibility Studies. Anim. Feed Sci. Technol..

[25] Alagawany M., Farag M.R., El-Hack M.E.A., Dhama K. (2015). The Practical Application of Sunflower Meal in Poultry Nutrition. Adv. Anim. Vet. Sci..

[26] Alagawany M., Attia A.I., Ibrahim Z.A., Mahmoud R.A., El-Sayed S.A. (2017). The Effectiveness of Dietary Sunflower Meal and Exogenous Enzyme on Growth, Digestive Enzymes, Carcass Traits, and Blood Chemistry of Broilers. Environ. Sci. Pollut. Res. Int..

[27] Senkoylu N., Dale N. (1999). Sunflower Meal in Poultry Diets: A Review. World’s Poult. Sci. J..

[28] Tavernari F., Albino L., Morata R., Dutra Júnior W., Rostagno H., Viana M. (2008). Inclusion of Sunflower Meal, with or without Enzyme Supplementation, in Broiler Diets. Rev. Bras. Cienc. Avic..

[29] Horvatovic M., Glamocic D., Zikic D., Hadnadjev T. (2015). Performance and Some Intestinal Functions of Broilers Fed Diets with Different Inclusion Levels of Sunflower Meal and Supplemented or Not with Enzymes. Rev. Bras. Cienc. Avic..

[30] Such N., Molnár A., Farkas V., Pál L., Husvéth F., Koltay I., Rawash M., Mezőlaki Á., Dublecz K. (2020). Feeding Two Single Strain Probiotic Bacteria and Wheat Bran Failed to Modify the Production Traits but Altered Some Gut Characteristics in Broiler Chickens. J. Cent. Eur. Agric..

[31] Dilger R.N., Onyango E.M., Sands J.S., Adeola O. (2004). Evaluation of Microbial Phytase in Broiler Diets. Poult. Sci..

[32] Kim J.W., Ndou S.P., Mejicanos G.A., Nyachoti C.M. (2017). Standardized Total Tract Digestibility of Phosphorus in Flaxseed Meal Fed to Growing and Finishing Pigs without or with Phytase Supplementation. J. Anim. Sci..

[33] Yu S., Cowieson A., Gilbert C., Plumstead P., Dalsgaard S. (2012). Interactions of Phytate and Myo-Inositol Phosphate Esters (IP1-5) Including IP5 Isomers with Dietary Protein and Iron and Inhibition of Pepsin. J. Anim. Sci..

[34] Cowieson A.J., Wilcock P., Bedford M.R. (2011). Super-Dosing Effects of Phytase in Poultry and Other Monogastrics. Worlds Poult. Sci. J..

[35] Lee S.A., Nagalakshmi D., Raju M.V.L.N., Rama Rao S.V., Bedford M.R. (2017). Effect of Phytase Superdosing, Myo-Inositol and Available Phosphorus Concentrations on Performance and Bone Mineralisation in Broilers. Anim. Nutr..

[36] Paul S.S., Rao S.V.R., Chatterjee R.N., Raju M.V.L.N., Prakash B., Yadav S.P., Kannan A., Reddy G.N., Kumar V., Kumar P.S.P. (2025). Evaluation of Phytase Super Dosing and Its Combinations with Other Additives as an Alternative to Antibiotic Growth Promoters in Broiler Chickens Reared in a Deep Litter System. Front. Anim. Sci..

[37] dos Santos T.T., Masey O’Neill H.V., González-Ortiz G., Camacho-Fernández D., López-Coello C. (2017). Xylanase, Protease and Superdosing Phytase Interactions in Broiler Performance, Carcass Yield and Digesta Transit Time. Anim. Nutr..

[38] Svihus B. (2011). The Gizzard: Function, Influence of Diet Structure and Effects on Nutrient Availability. Worlds Poult. Sci. J..

[39] Hemati Matin H.R., Shariatmadari F., Karimi Torshizi M.A., Chiba L.I. (2016). In Vitro Bile Acid-Binding Capacity of Dietary Fibre Sources and Their Effects with Bile Acid on Broiler Chicken Performance and Lipid Digestibility. Br. Poult. Sci..

[40] Choct M., Annison G. (1992). Anti-nutritive Effect of Wheat Pentosans in Broiler Chickens: Roles of Viscosity and Gut Microflora. Br. Poult. Sci..

[41] Ravindran V., Selle P.H., Ravindran G., Morel P.C.H., Kies A.K., Bryden W.L. (2001). Microbial Phytase Improves Performance, Apparent Metabolizable Energy, and Ileal Amino Acid Digestibility of Broilers Fed a Lysine-Deficient Diet. Poult. Sci..

[42] Rutherfurd S.M., Chung T.K., Thomas D.V., Zou M.L., Moughan P.J. (2012). Effect of a Novel Phytase on Growth Performance, Apparent Metabolizable Energy, and the Availability of Minerals and Amino Acids in a Low-Phosphorus Corn-Soybean Meal Diet for Broilers. Poult. Sci..

[43] Lemme A., Ravindran V., Bryden W.L. (2004). Ileal Digestibility of Amino Acids in Feed Ingredients for Broilers. Proc. World’s Poult. Sci. J..

[44] Ullah Z., Ahmed G., Nisa M.U., Sarwar M. (2016). Standardized Ileal Amino Acid Digestibility of Commonly Used Feed Ingredients in Growing Broilers. Asian-Australas. J. Anim. Sci..

[45] Wolfrum S., Siegert W., Rubio-Cervantes I., Liebhold T.M., Feuerstein D., Camarinha-Silva A., Rodehutscord M. (2025). Interactions between Dietary Phytate Concentration and Phytase Level on Phytate Degradation and Amino Acid Digestibility in Broiler Chickens. Poult. Sci..

[46] Krieg J., Siegert W., Berghaus D., Bock J., Feuerstein D., Rodehutscord M. (2020). Phytase Supplementation Effects on Amino Acid Digestibility Depend on the Protein Source in the Diet but Are Not Related to InsP6 Degradation in Broiler Chickens. Poult. Sci..

